# Extraction of high quality and high yield RNA from frozen EDTA blood

**DOI:** 10.1038/s41598-024-58576-9

**Published:** 2024-04-15

**Authors:** Long T. Nguyen, Carol A. Pollock, Sonia Saad

**Affiliations:** Renal Medicine, Kolling Institute, University of Sydney, Camperdown, Australia

**Keywords:** Isolation, separation and purification, Biomarkers

## Abstract

Peripheral blood RNA profiling, which can reveal systemic changes in gene expression and immune responses to disease onset and progression, is a powerful tool for diagnosis and biomarker discovery. This technique usually requires high quality RNA, which is only obtainable from fresh blood, or frozen blood that has been collected in special RNA-stabilisation systems. The current study aimed to develop a novel protocol to extract high quality RNA from frozen blood that had been collected in the conventional EDTA tubes. We determined that thawing EDTA blood in the presence of cell lysis/RNA stabilisation buffers (Paxgene or Nucleospin) significantly improved RNA quality (RIN) from below 5 to above 7, which to date has not been shown possible. The EDTA-Nucleospin protocol resulted in 5 times higher yield than the EDTA-Paxgene-PreAnalytix method. The average RIN and mRNA expression levels of five different genes including 18 s, ACTB, MCP1, TNFa and TXNIP using this protocol were also indifferent to those from Paxgene blood, suggesting similar RNA quality and blood transcriptome. Moreover, the protocol allows DNA to be extracted simultaneously. In conclusion, we have developed a practical and efficient protocol to extract high quality, high yield RNA from frozen EDTA blood.

## Introduction

Blood is a near unlimited source of biological materials to be used for disease diagnosis and monitoring. Blood is routinely collected from both healthy individuals and patients due to the minimal invasiveness of the procedure. Additionally, in research, blood-derived proteins, metabolites, DNA and RNA can all be used for biomarker discovery and mechanistic investigation of different diseases. For example, blood RNA profiling has been used to identify candidate biomarkers for classification and prognostication of diabetes and complications^1–4^, pulmonary disease^5^, cardiovascular disease^6,7^, neurological diseases^8,9^, and cancer^10,11^. However, RNA is highly susceptible to degradation due to its relatively unstable structure and the ubiquitous presence of RNases in the environment. Suboptimal collection and storage conditions of blood can lead to haemolysis and release of RNases from red blood cells, resulting in RNA degradation. Degraded RNA is generally not recommended for library construction in next generation sequencing, especially when coding regions are of primary interest.

Ethylenediaminetetraacetic acid (EDTA)-coated tubes are the conventional containers for blood collection, which although can be used to extract intact RNA from fresh blood, does not contain RNA stabilising chemicals to protect RNA from degradation during freezing and thawing. It is well-known that RNA extracted from frozen EDTA blood not only has a low yield but is also highly fragmented^12,13^. In most cases, blood is collected at the clinic but processed centrally later elsewhere. Freezing can temporarily deactivate RNases but freeze–thaw cycles can result in uncontrolled haemolysis and release of RNases, making RNA degradation inevitable. As such, legacy blood samples that were collected in EDTA tubes are generally deemed not suitable for transcriptional profiling.

RNA stabilisation systems such as Paxgene (BD Biosciences) or Tempus (Thermofisher Scientific) are now available to address the issue. In this method, whole blood needs to be mixed with specific buffers at the time of collection to lyse blood cells and inhibit RNases in a well-controlled manner. RNA is precipitated from the solution in these buffers and therefore preserved intact for later purification. However, there have been reports showing that RNA extracted from Paxgene tubes contained DNA contamination or inefficient in PCR reaction^13,14^. Moreover, as these kits are expensive and incompatible with other biochemical assays, they are usually not routinely stocked for clinical use unless being specified as part of a clinical trial. This means to extract intact RNA for transcriptional profiling, prospective human studies are generally required, with blood being drawn for storage in tubes suitable for RNA extraction which involves extensive commitment and inevitable cost that otherwise could be avoided. The recruitment of new patient cohorts also means that the use of historic biobanks are not optimally utilised and current and past data cannot be correlated, thus limiting data interpretation.

For standard RNA-seq library preparation, the recommended RNA amount and RNA integrity (RIN) is 500 ng and 7–8 respectively. Higher RINs mean that RNA is less degraded. RNA extracted from frozen EDTA blood using traditional methods (e.g. Trizol) tends to have RIN of < 4^12,15,16^. Beekman and colleagues attempted to overcome this problem by transferring EDTA blood to PAXgene tubes followed by RNA extraction using a PAXgene-compatible kit. This approach was shown to increase RNA yield and integrity (RIN = 6)^12^, which may be acceptable for certain applications such as microarray^12^. However, as the impact of suboptimal RIN on RNA sequencing is unclear, RNA with RIN > 7 is still requested by Illunima and most sequencing service providers, particularly when mRNA is of the primary interest.

Several groups have attempted to improve the quality of EDTA blood-derived RNA to meet mRNA-Seq standards^15,16^. However, RIN = 6 appears to be the highest achievable value and thus still remain suboptimal. The ability to extract high-quality RNA from frozen EDTA blood will allow researchers to simplify and standardise the sample preparation procedure to achieve the highest efficiency and reproducibility in RNA biomarker research. Legacy cryopreserved blood samples across the world can be used and data generated from the same samples/cohort can be correlated to gain novel insights into different diseases and therapeutic effects. This study aimed to compare different RNA extraction protocols to identify the determining factors to extract high quality RNA from frozen EDTA blood for mRNA-seq.

## Results

### The effects of incubation and extraction kits in the EDTA-to-PAXgene transfer method on blood RNA quantity and quality

First, we assessed the impact of using different kits to extract RNA from EDTA blood that was transferred into Paxgene tubes before extraction. As shown in Table 1, EDTA-Paxgene-PreAnalytix protocol showed a 36% increase in yield compared to the PAXgene PreAnalytix (PP) control method. However, RIN was approximately halved (*P* < 0.05) and A260/230 was significantly lower (*P* < 0.01), suggesting the expected poor quality. If the Magmax extraction kit was used instead of PreAnalytix, we found no change in yield, a slightly less drop in RIN (*P* < 0.01) but a much more significant reduction in A260/230 (*P* < 0.01).

**Table 1:** RNA extraction following blood transfer from EDTA to Paxgene tubes.

Protocol			Yield (μg RNA/ml blood)	% PP-Ctrl	RIN	260/280	260/230
Paxgene Ctrl		PreAnalytix	1.00 ± 0.31	100.00	8.00 ± 0.2	2.14 ± 0.06	1.54 ± 0.04
EDTA blood transfer to Paxgene tube	Kit	PreAnalytix	1.38 ± 0.48	136.76 ± 5.74*	4.00 ± 0.3**	2.15 ± 0.08	0.94 ± 0.11*
		Magmax	1.04 ± 0.23	107.10 ± 9.56	4.55 ± 0.25**	2.15 ± 0.05	0.39 ± 0.14**
	Incubation	2 h	0.85 ± 0.03	123.07 ± 3.65	4.87 ± 0.30**	2.08 ± 0.01	0.59 ± 0.21**
		overnight	1.37 ± 0.22	104.83 ± 17.31	3.97 ± 0.15**	2.22 ± 0.01	0.57 ± 0.11**
	Refreeze	No	1.21 ± 0.24	107.27 ± 11.98	4.28 ± 0.23**	2.15 ± 0.04	0.66 ± 0.17**
		Yes	0.90 ± 0.05	83.60 ± 6.47	4.70 ± 0.57**	2.16 ± 0.06	0.42 ± 0.03**

EDTA blood was thawed on ice for at least 2 h before transferring to Paxgene tubes. Data are expressed as Mean ± SEM. N = 2.

*P < 0.05, **P < 0.01.

Next, we questioned if varying sample incubation time before RNA extraction would make an impact on RNA quantity and quality. The result showed that there were no improvements in any of the measurements when the blood was incubated for either 2 h or overnight before extraction. Refreezing the blood in − 80 °C also had no effect.

### The effect of cell lysis/RNA stabilisation buffer

As RNA integrity remained low despite blood was transferred to Paxgene system after thawing, we tried to address the key question as to whether RNA degradation occurred during cryopreservation or during the thawing process. If degradation had already occurred during cryopreservation, then attempts to improve RNA integrity would not be fruitful. By adding Paxgene additives to EDTA tubes during thawing instead of vice versus, RIN was significantly improved to 7.3 ± 0.14, while RNA yield remained similar (*P* < 0.01, Table 2).

**Table 2:** The effect of lysis buffers in Paxgene and Nucleospin kits on blood RNA degradation.

Blood tube	Pretreatment	Incubation	Extraction	%PP-Ctrl	RIN
EDTA	Thaw on ice then transfer to Paxgene tube	RT (2 h)	Pre-AnalytiX	136.76 ± 5.74	4.00 ± 0.3
EDTA	Add Paxgene solution to EDTA tube then thaw on ice	RT (2 h)	Pre-AnalytiX	148.77 ± 33.79	7.30 ± 0.14**
EDTA	Add Nucleospin lysis buffer then thaw on ice	RT (15 min)	Nucleospin	649.77 ± 72.51**	8.00 ± 0.21**

Data are expressed as Mean ± SEM. N = 2.

**P < 0.01.

To confirm that this effect is not specifically attributed to Paxgene additives but to the reverse addition of cell lysis/RNA stabilisation buffer, we tested the Nucleospin blood RNA extraction from Macherey–Nagel. Similar to the EDTA-Paxgene reverse transferring method followed by PreAnalytix extraction kit, adding Nucleospin lysis buffer to EDTA blood before thawing resulted in a significantly improved RIN of 8 ± 0.21 (*P* < 0.01). At the same time, RNA yield was also 5 times higher than EDTA-PP method (*P* < 0.01, Table 2). This protocol is referred to as EDTA-mixed thawing-Nucleospin (EmN).

### Blood RNA yield and RIN using reference and novel protocols

**Figure 1:**
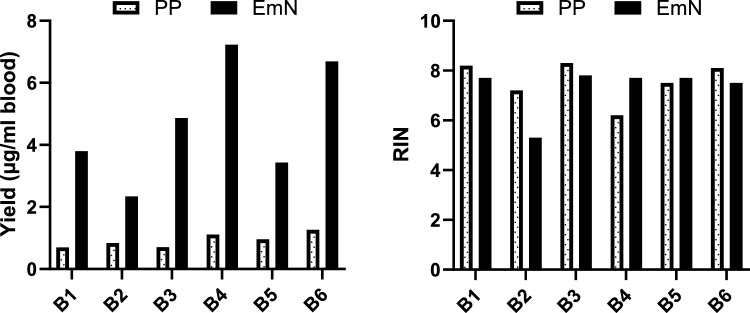
Individual blood RNA yield and RIN using PAXgene-PreAnalytix (PP) control protocol and EDTA-mixed Nucleospin method (EmN). PP and EmN used 2.5 and 1.3 ml of blood respectively.

To validate the utility and efficacy of the EmN protocol, we collected and processed blood from six healthy volunteers using the reference protocol (PAXgene tube followed by PreAnalytix blood RNA kit) and EmN. As can be seen from Table 3, the average RNA yield was 0.9 ± 0.2 μg/ml blood for the standard protocol and 4.7 ± 1.9 μg/ml for the EmN protocol, (*P* < 0.001). The A260/280 and A260/230 ratios were also the same (Table 3). Importantly, RINs were similar between the two groups (7.6 for PP and 7.3 for EmN). Both protocols produced RNA with RIN above the requirement for RNA-seq from 5/6 samples (Fig. 1).

**Table 3:** Blood RNA quantity and quality using EmN and PAXgene-control protocols.

Protocol	Yield μg/ml blood	RIN	260/280	260/230
PP	0.9 ± 0.2	7.6 ± 0.8	2.1 ± 0.0	1.7 ± 0.1
EmN	4.7 ± 1.9	7.3 ± 1.0	2.1 ± 0.0	1.7 ± 0.2

Data are expressed as Mean ± SEM. N = 6.

### Expression of blood genes using reference and novel protocols

**Figure 2:**
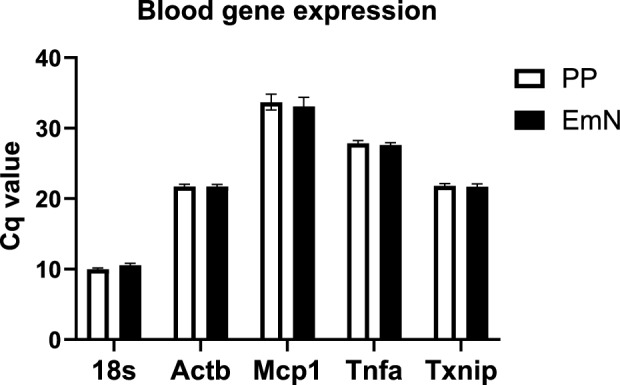
mRNA expression of reference and target genes in human blood processed by the standard protocol (PP) and novel protocol (EmN).

We next compared the mRNA expression of five common genes in the blood using the two different protocols. EmN showed nearly identical Ct values across all the five different genes including 18 s, ACTB, MCP1, TNFa and TXNIP, suggesting similar transcriptomic expression profiles between EmN and PP the reference methods (Fig. 2).

### Co-extraction of RNA and DNA

EDTA blood collection tubes are available in different sizes and the most common size for human blood is 3 or 4 ml. Since this is more than what is needed for RNA extraction (1.3 ml), the unused blood will be wasted. We therefore attempted lysing blood cells with the lysis buffer from Nucleospin blood RNA kit then using other components from Nucleospin blood DNA kit to co-extract DNA. As can be seen from Table 4, DNA yield was 4.3 ± 1.2 μg/ml blood, which is more than the general input requirement of 500 ng for DNA profiling. A260/280 was 2.0 while A260/230 was approximately 1.1.

**Table 4:** Blood DNA quantity and quality when coextracted with RNA.

	Yield (μg)	Yield (μg/ml blood)	260/280	260/230
EmN	8.5 ± 2.4	4.3 ± 1.2	2.0 ± 0.0	1.1 ± 0.1

## Discussion

Blood is the most frequently tested biological material for disease diagnosis and monitoring. Proteomic, metabolomic and transcriptomic profiling of blood can reveal fundamental changes that are associated with a particular disease but remain asymptomatic, allowing early intervention to occur. While proteomic and metabolomic studies can be done on plasma/serum that is fractionated from whole blood collected in EDTA tubes, transcriptomic profiling requires intact RNA, and extraction from EDTA cryopreserved blood is a challenge. Overall, the result suggests that the thawing of EDTA blood was the key process to RNA stabilisation or degradation, in which changing the extraction method post thawing did not improve RNA quality. Only by adding RNA stabilising buffer to EDTA blood during thawing, RNA integrity could be improved to the same level as the standard PAXgene PreAnalytix method. Lastly, Nucleospin was shown to give a 5 times higher yield of RNA compared to PAXgene-based kits.

Blood has a complex cellular composition comprising of 1% white blood cells (WBC), which contain the RNA of interest and more than 99% of RBCs. As stated previously, RBCs contain high amounts of RNase that is released upon cell lysis. In addition, immature reticulocytes contain high levels of globin mRNA that are non-specific and can interfere with the detection of WBC-derived mRNA. To protect the mRNA for later extraction, fresh blood is traditionally treated with RBC lysis buffer while intact WBCs are pelleted for storage^17^. This approach minimizes presence of RNase and adhesion of RNA to haemoglobin and cell debris to increase RNA yield and integrity but is not applicable to thawed EDTA blood since WBCs are already destroyed due to freezing and thawing. In addition, WBCs removal demand skill, specialised equipment, and time, which may not be available in routine blood collection clinics. It has been demonstrated that freezing techniques have a significant impact on cell survival and gene expression^18^. Similarly, pre-cryopreservation technical variation due to equipment and handling may induce variability in RNA quality and expression profile at the time of extraction. In comparison, EDTA blood is stored in − 20 °C or − 80 °C shortly after collection, hence reduces variability in RNA quality.

In RNA stabilising systems such as Tempus/Paxgene, whole blood will be lysed immediately in the presence of RNase inhibitors. RNA is selectively precipitated and therefore protected from degradation. In our study, the RNA yield using Paxgene was relatively low (0.9 μg/ml blood or 2.25 μg/2.5 ml blood/extraction), likely because RNA is trapped in the cell debris pellet, the size of which varies from sample to sample. According to our experience, smaller pellets usually correlate with higher RNA yield and increased mixing did not increase the resolution of the pellet. Although RNA yield from PAXgene is sufficient for RNA sequencing, having enough RNA for further experiments such as qRT-PCR could be problematic. In addition, because PAXgene tubes are 15–20 times more expensive than EDTA tubes, they are only purchased if RNA sequencing is planned, and not generally stocked in the clinics. Being able to extract high quality RNA from frozen EDTA blood will allow routine RNA extraction from blood for biomarker discovery and disease diagnosis to become more practical.

In this study, blood samples were generally frozen shortly after collection so the effects of the time before freezing on RNA quality was not investigated. However, it has been demonstrated that blood samples stored in 4 °C for a longer time tend to yield more degraded RNA^19^. In contrast to the general belief that freezing is the defining factor of RNA quality^18^, the fact that RIN was improved to 7–8 (equivalent to that of PAXgene control protocol) simply by changing thawing condition suggests that freezing does not have a major effect on RNA degradation and that EDTA blood RNA remains mostly intact during freezing and cryopreservation Transferring thawed EDTA bloods to RNA-stabilising buffers can improve RNA integrity compared to direct extraction from EDTA tubes. However, RIN = 6 was the highest average RIN reported and thus remains suboptimal^12,15,16^. Recently, Yamagata attempted thawing EDTA blood on aluminium blocks at room temperature to minimize RNA degradation^16^ but was unsuccessful in improving RIN to above 7, which as previously discussed is required for library construction in RNA sequencing. This suggests that adjusting temperature is important but not adequate to protect against blood RNA degradation, which was likely due to the absence of RNA stabilising reagents. Indeed, when RNA stabilising reagents (mixed in lysis buffer) was added to EDTA tubes while the blood was still frozen instead of waiting for it to thaw, RNA integrity was improved to the level of the control method (RIN > 7). This indicates the inhibition of RNases that had been gradually released, which were otherwise reactivated following the rising temperature. Such improvement did not depend on the type of buffer (Paxgene or Nucleospin) that was used, suggesting the two buffers were equally effective in inhibiting RNA degradation.

It is noteworthy that several total RNA sequencing kits (e.g. TruSeq) can be used to assess low quality RNA (RIN < 7)^20^. Although total RNA-seq allows detection of non-coding RNAs, its accuracy and specificity for coding mRNAs is low. This is because: (1) mRNA accounts for only about 3–7% of the mammalian transcriptome and (2) total RNA-seq relies on ribosomal RNA removal instead of Poly(A) affinity selection that is mRNA-specific. This is usually not a major problem if RNA is intact. However, if RNA is degraded, this can interfere with PCR reactions and skew gene expression profile due to disproportional amplification of sequencing targets. In the worst case, certain mRNA can be completely lost or degraded during upstream workflow steps, which increases the risk of false-negative results. As such, regardless of whether total RNA-seq or mRNA-seq is used, a high RIN is always preferable. Moreover, compared to total RNA-seq, library preparation can be performed with smaller sample sizes for mRNA-Seq and sequencing depth can be increased, which significantly improves sequencing efficiency and reduces cost.

In comparing the Paxgene/PreAnalytix control method and EDTA-Nucleospin, the latter has been shown to improve RNA yield by 5 times^16^. The same result was achieved in the current study, suggesting that Nucleospin is indeed much more efficient in RNA recovery. This is critical when the amount of blood for RNA extraction is limited, which is generally the case if blood is historically stored from clinical trial participants. In addition, we found Nucleospin protocol is much simpler. It has only 8 steps and takes ~ 45 min to complete, compared to PreAnalytix kit which consists of 21 steps and can require up to 2 h of processing time. Regarding RNA quality and expression, our improved protocol showed equivalent RIN and identical expression levels of 5 reference genes, suggesting data of the two methods are likely comparable, although whole transcriptomic sequencing is required to fully assess similarity of the two expression profiles.

A potential drawback of the mixing-before-thawing method is that blood in a whole EDTA tube generally needed to be entirely thawed^16^, therefore cannot be saved for other purposes. In most cases this should not be a problem because most blood-based assays such as ELISA and metabolite measurement can only be done on serum/plasma, which requires separation from fresh blood before cryopreservation. Frozen whole blood is primarily used for DNA extraction and as we demonstrated in this study, it is also possible to concurrently extract RNA and DNA after the blood is treated with cell lysis buffer from Nucleospin blood RNA kit. A proportion of the lysed blood (up to 1.3 ml) will continue to be used for RNA extraction by the same kit, while the rest can be used for DNA extraction using Nucleospin blood DNA kit. This dual extraction method showed sufficient yield for next-gene sequencing of both RNA and DNA. Although A260/230 of the extracted DNA was relatively low, this could be further addressed by running the samples through Bio-spin columns multiple times until a desirable A260/230 is achieved.

In summary, we have optimised a protocol to extract high quality and quantity RNA from EDTA tubes to study gene expression. In addition, Nucleospin blood RNA and DNA kits can be used together to isolate both RNA and DNA from the same extraction. Should the new method be adopted, all legacy EDTA blood can be utilised for gene expression profiling and future studies no longer need to rely on expensive RNA stabilising systems for blood storage.

## Methods

### Patients

Participants of either sex aged between 18 and 75 years of age were included. Informed consent was obtained from all participants involved in this study. The study was carried out in accordance with relevant guidelines and regulations was approved by the human Ethics committee at Royal North Shore Hospital (Ref: HREC/17/HAWKE/471). Blood from a volunteer (~ 10 ml/collection) was used to optimise RNA quantity and quality. Then a cohort of six healthy individuals were recruited to compare the novel method with the standard protocol using PAXgene system.

### Sample collection and storage

For each participant, blood was collected by a clinical nurse in one PAXgene tube (2.5 ml) and two EDTA tubes (4 ml/tube) respectively. PAXgene tubes (Becton–Dickinson) were mixed thoroughly and stored in − 20 °C overnight followed by long-term storage in − 80 °C freezer. Blood collected in EDTA tubes (Sarstedt) were directly stored in − 80 °C freezer. Samples were kept at − 80 °C for 2–3 months until RNA extraction.

### RNA extraction strategies

For RNA extraction from original Paxgene tubes, samples were thawed on ice for at least 2 h and PAXgene Blood RNA Kit (PreAnalytix, Hombrechtikon, Switzerland) was used to extract blood RNA according to the manufacturer’s manual. This method is referred to as Paxgene Control or PP. For EDTA tubes, we had two strategies to optimise the RNA quantity and quality. The first was to replicate and optimise a reference protocol based on transferring EDTA blood to PAXgene tubes before extraction. Several conditions were then tested including (1) Different extraction kits: PAXgene Blood RNA Kit (PreAnalytix, Hombrechtikon, Switzerland) versus MagMAX blood RNA Isolation Kit for PAXgene (Thermofisher Scientific, MA, USA), (2) different post-transfer incubation time: 2 h versus overnight, and (3) same day extraction vs refreezing the samples for later extraction. The second strategy was to optimise the Nucleospin blood RNA extraction kit (Macherey–Nagel, Westfalen, Germany), which was primarily meant to be used for fresh blood but has been shown to produce a much higher yield of RNA compared to other protocols using frozen blood^15,16^.

### DNA extraction

4 ml of blood was thawed using lysis buffer in Nucleospin blood RNA kit then 2.0 ml of blood was moved into a new centrifuge tube for DNA isolation. Blood DNA was extracted using Nucleospin Blood DNA Midi Kit according to the manufacturer’s instruction, followed by salt clearance using Micro Bio-Spin™ P-30 Gel Columns, Tris Buffer (Bio-rad, CA, USA).

### RNA/DNA quantity and quality check

RNA concentration and quality were measured by TapeStation 4200 (Agilent, CA, USA). DNA concentration was measured by Nanodrop 100. A260/A230 and A280/260 were measured using Nanodrop.

### Real-time qPCR

500 ng of RNA from each sample was used for cDNA synthesis using iScript™ gDNA Clear cDNA Synthesis Kit (Bio-rad, CA, USA) and RT-PCR was run using iTaq™ Universal SYBR® Green Supermix according to the manufacturer’s instructions. In order to compare the mRNA expression profiles of the blood processed by the reference protocol and the novel method, two reference genes including 18 s and β-actin (ACTB) were examined. In addition, Monocyte Chemoattractant Protein 1 (MCP1), Tumour necrosis factor alpha (TNF-α) and Thioredoxin-Interacting Protein (TXNIP) were tested as genes of interest as they are involved in inflammation and diabetes (ref).

### Statistics

For protocol optimisation, each condition was tested in duplicate on two different days using blood from the same volunteer, and data are expressed as Mean ± SEM. For validation, a human cohort of n = 6 was used and data are expressed as Mean ± SD. Unpaired t-tests were used for each comparison and *P* < 0.05 was considered significant.

## Data Availability

The datasets generated during and/or analysed during the current study are available from the corresponding author on reasonable request.
